# Book Reviews

**Published:** 1810-04

**Authors:** 


### CRITICAL ANALYSIS OF THE RECENT PUBLICATIONS IN THE DIFFERENT BRANCHES OF PHYSIC, SURGERY, AND MEDICAL PHILOSOPHY.

---

*An Enquiry into the Nature, Causes, and Cure of* HYDROTHORAX, *illustrated by interesting Cases, and many living Examples of the Success of the Mode of Treatment recommended.* By L. MACLEAN, M. D. 8vo. pp. 519. London, 1810.

IT has very justly been observed that the advancement of Science has been much accelerated by a predilection which many of its Professors have entertained for some one of its branches. The powers of the mind thus concentrated on one subject, must necessarily atchieve more than when directed to the contemplation of a wide and extensive range of objects. Impressed with such a conviction, we are ever happy to congratulate the medical world upon the acquisition of any new work, when it contains a history of the labours of a medical practitioner, exclusively devoted to the investigation of one disease. The publication of the volume which we now beg leave to announce to our readers, is of this nature; and its author was first induced to direct his attention to the disease termed *hydrothorax*, because (as he informs us) it has been hitherto, in a great measure overlooked, or so slightly touched upon, as if it claimed no more notice than the common disorders of every season; yet, whether we regard the frequency of its occurrence, the formidable aspect it assumes, the extreme sufferings of the patient, or the almost uniformly fatal termination to which it leads, few demand investigation more than dropsy of the chest. He, however, with much candour, acknowledges in his Preface, that "If the lovers of novelty should look for new remedies, they will be disappointed; but they will find old medicines, or those in common use, applied in different forms and combinations, with different views, and he presumes to hope, with a degree of success unparalleled in the history of the disease:—they will find that in every instance it may be relieved, that in a considerable number it may be kept in check for months, and years, with long intervals of repose, or comparative ease and comfort to the patient; and that in some, it may be perfectly and permanently cured, even under the most unpromising circumstances. This, it is presumed, is more than can be accomplished, in the present state of our knowledge, from any mode of treatment hitherto adopted." If such be the success which has attended his inquiry into the cause of this disease, he has certainly atchieved much for the practice of medicine, by removing one of its most lamentable *opprobria*, and we we may confidently assure him, that the lover of novelty, so far from requiring any apology to appease his indignation, will terminate the perusal of the book with infinite satisfaction and delight; whether, however, an object so desirable has actually been obtained, we shall endeavour to ascertain, by a candid and impartial inquiry. The objects which the Treatise comprises, may be arranged under four heads, viz. I. The Nature and Symptoms of the Disease. II. Its various Causes. III. Method of Cure. IV. Cases illustrative of the plan of Treatment. An analysis of each of these divisions, we shall in succession offer to our readers.

After having taken a view of the different definitions of hydrothorax, as given us by Sauvage, Vogel, Sagar, and Cullen, our author proposes to us an essential character, which appears to him less equivocal than those of the foregoing authors, which contain a train of symptoms which are neither essential to the disease, nor inseparable from it; but so protiform and insidious are the symptoms, that we fear he has succeeded but little better than preceding nosologists in framing an unequivocal definition. He then enters at some length, into an investigation of the *precursors*, or early signs, by which the disease may be detected in its incipient stages; these are valuable considerations for the medical practitioner, and we regret that our author's experience has not enabled him to throw more light upon this obscure part of the subject, for whatever merit we may be inclined to give him for his judicious selection, and arrangement of the *precursors* which have been already detailed by other authors, he has certainly added no new information which might enable us more easily and more infallibly to detect the early existence of this "*anguis in herba.*" He next proceeds to the enumeration of the *particular* symptoms, in which he traces their origin, order of precedency, progress, and termination. The derangement of the vital functions is evinced by an impeded respiration and palpitation of the heart, with a corresponding irregularity of the pulse; there is a difficulty, or impossibility of retaining the recumbent posture, especially when the patient is placed on one side; the aspect of the countenance is pallid and livid, and the urine is scanty and high coloured; œdematous swellings, with cold and senseless extremities appear, and the patient is often suddenly aroused from sleep by a most distressing sense of suffocation. Such he informs us may be considered the leading characteristic symptoms of the disease; although many others, not essential to it, very frequently occur, such as cough, expectoration of blood, erratic pains in the region of the chest, external swelling, and fluctuation of water.

Our attention is next directed to the diagnostic symptoms of *dropsy of the pericardium* and of *hydrothorax*; here our author has rather convinced us of the intimate and extensive knowledge which he possesses of the opinions of other writers upon this subject, than afforded us any original observations of intrinsic worth; for we cannot be so unjust to our readers, as to omit observing, that our own experience concurs with the testimony of other practitioners, in inducing us to believe that every symptom which is adduced as pathognomonic in *hydrops pericardii* occurs differently modified, and variously combined with others, as well in hydrothorax as in every morbid alteration in the structure of the heart, or any thoracic viscus; the other diseases with which hydrothorax may be confounded are then detailed; but as the experienced practitioner will find no difficulty in easily distinguishing them, we shall abstain from any farther observation. The prognosis is next considered, which we are informed, must be entirely guided by our knowledge of the cause of the disease; an eulogy on the importance of dissection, in advancing our knowledge of this disease, then terminates the first division of the subject.

Amongst the occasional causes of dropsy, our author very properly regards every circumstance which tends to depress or destroy the vital energy of the body; and he considers, that the inordinate potation of porter, ale and beer, more frequently excites the disease *hydrothorax*, than the abuse of other fermented liquors; this he explains by attributing to them a two-fold mode of operation, by undermining in common with other intoxicating liquors the powers of the constitution, and by favouring an accumulation of fat without imparting a proportionate increase of strength; hence those organs essential to life, are overwhelmed with fat, and are consequently inadequate to perform their proper functions.

We are happy to see that our Author considers that the different species of fermented liquors, individually produce different effects on the organization of the body. Whether this is explicable upon the supposition that they impart different proportions of *oxygen* and *carbon* to the blood, we shall not pretend to decide; the fact itself is sufficiently evident, and a farther investigation of the subject, conducted on the solid basis of experiment, would afford many valuable materials for the speculations of the physiologist, and throw considerable light upon the theory of *Dietetics*.

With respect to an accumulation of fat producing Hydrothorax, we cannot but entertain some doubts; the numerous dissections the writer of this article has seen, in which the greater proportion of hydrothoracic subjects exhibited no accumulation of fat about the vital organs, induce him to believe it is very rarely the cause of this disease.

Dr. Darwin's celebrated theory of the *Retrograde action of the absorbents* is then examined at some length, the existence of which he concludes has neither been proved by experiment or rational induction.

The influence of the biliary secretion in the production of dropsy, he considers as not sufficiently understood; it is generally supposed, continues he, that a diseased liver is concerned only in producing dropsy by preventing the free return of the blood of the *vena portarum* and *inferior cava* to the heart, but its influence is exerted earlier, and is far more extensive than is generally imagined.

gined. He conceives that bile, although an active stimulus to the bowels, is a sedative to other organs, and illustrates his opinion by adducing as an example the torpor and languor which pervade the bodily and mental functions in jaundice. This being conceded, he concludes that the urinary secretion and lymphatic absorption will be always more or less diminished, and that a corresponding tendency to dropsy will ensue.

We next proceed to the consideration of his peculiar plan of cure; and as this division of the subject is not only the most interesting to the medical practitioner, but the most important and decisive in establishing the credit and character of its Author, we shall endeavour to exhibit to our Readers some of the most prominent features of his treatment. He does not profess, as we before stated, to introduce to our notice any new medicines, but proposes to effect all that is desirable by a novel and happy combination of usual remedies. We must here be allowed to observe, that the fashion of combining medicines in every proportion and degree, has more or less obtained amongst the disciples of different schools; we sometimes find the prescription of the physician consisting of twenty compound articles, whilst the same disease is frequently combated with equal success by another physician by the exhibition of a simple herb. In general, the idea of combining a number of remedies with the hopes of the compound partaking of the virtues of each constituent is altogether fallacious; and it is our opinion, that the physician who prescribes with the greatest simplicity, prescribes the best, although the maxim is, doubtless, liable to many exceptions. We do not, however, mean to depreciate the merits of our Author's plan by any preconceived opinions. He prefaces the subject by some general observations, in which he offers several valuable remarks on the different effects of diuretic medicines; "there is," says he, "such a material difference in their mode of operation, that there appear just grounds for dividing them into three different classes, viz.

1. Such as act chiefly, if not solely, on the kidneys.
2. Such as act at the same time on the kidneys, the absorbents, the exhalents, and other secretions and excretions.
3. Such as act solely on the absorbents.

Under the first class, nitre is the only one which ought to be comprehended; under the second, by far the greater number of diuretics in daily use may be included; under the third class, says he, I know no substance of which I dare speak with confidence, except the fox-glove. "The practical inferences arising out of these facts and observations seem obvious," continues he, "for if by experience it be ascertained, that these substances all act differently, is it not reasonable to infer, that by blending and combining them according to their specific powers, a benefit is more likely to result than when separately administered, partially applied, and probably not to the organ which most needs their aid?" He tells us, that if the patient be advanced in years, or his strength exhausted by intemperance, a remedy should be selected of such combined powers as might answer every indication at once; a combination of foxglove, certain tonics, saline diuretics, and calomel in moderate doses, will be found the best in such cases; but if the disease occur in delicate subjects, without any organic affection, the digitalis alone will generally succeed; but its salutary effects will be promoted by tonics and moderate doses of the fixed vegetable alkaline salts. When it happens in fat, corpulent subjects, with a sluggish and inirritable fibre, such a combination as will produce the most extensive operation should be prescribed; he then descends into a consideration of particular remedies, which comprehends *blisters,* whose effects he considers important; *digitalis* in the form of an infusion of its leaves, the *squills,* which he thinks should always be combined with some other diuretic; *saline diuretics,* which though precarious and uncertain in their operation, in combination with other means, and largely diluted, will be found powerful auxiliaries; *crystals of tartar* he thinks should be used with caution, especially in thin, delicate habits, where the general strength is much impaired; for it excites the action of the lymphatics to such a degree, as not only to absorb the effused fluids, but also fat and muscle.

*Kali præparatum* he is inclined to prefer, in many cases, as more safe and certain in its operation, which will not only remove the water, but in combination with aromatics strengthen the digestive organs; its operation seems to be confined chiefly to a morbid state of the absorbents, since in health it produces but little diuretic effect; *broom ashes* owe their virtues evidently to this substance contained in them; the diuretic virtues of this alkali are promoted by the nitrous and other æthers, by the turpentines and certain balsams.

*Æther* and its preparations he considers as excellent auxiliaries in this disease; they afford immediate relief in distressing respiration; the *nitrous æther* he considers as possessing less antispasmodic, but greater diuretic powers; the *resins and resinous balsams* should be exhibited in combination with squills and crystals of tartar, and will be successful principally in those cases where there is torpor of the liver; *laxatives* should always be employed, when the body is not naturally open; and such as are known to act at the same time on the kidneys; a combination of calomel and crystals of tartar may be given at night, and an infusion of senna the following morning; *calomel and other preparations of mercury,* our author considers more salutary in their agency than is generally supposed; "ample experience, says he, has enabled me unequivocally to ascertain their efficacy." Much will depend however on the quantity and mode of exhibition; if any of its oxyds or saline combinations be introduced into the stomach, in their crude, unqualified state, they will tend rather to defeat, than to promote our wishes. He prefers calomel, because it is less liable to variation of strength, and is more readily blended with other substances; substances; its virtues will be always materially increased by previous trituration with some saline substance; and he asserts that two grains thus prepared and blended, will produce a more certain effect, than ten in the ordinary way, and without any, or comparatively with very little, pain or irritation. Whenever there is reason to suspect any derangement in the lymphatic functions, he recommends us occasionally to stimulate the bowels with mercurial purgatives, and at the same time, to charge the habit with it by giving one or two grains of calomel every night. *Blood-letting.*—Cases he thinks sometimes occur, where this operation is absolutely necessary, an example of which is, when the congestion and the accumulation of blood in the right side of the heart, and in the head, (owing to the interrupted circulation through the lungs) threaten suffocation.

The tonics which should be employed after the removal of the water, must in great measure be directed by the circumstances of the case; in general, the light infusions of gentian and columbo, in combination with steel and myrrh, are to be preferred; but if there is a hard dry cough with viscid expectoration, he recommends the exhibition of steel in conjunction with the balsam; *opiates* in this disease he thinks admissible, when the cough is very urgent.

*Elaterium*, our author has but seldom prescribed, "yet," says he, "from the few trials which I have made, I am inclined to think that it might be found useful in certain cases, cautiously administered in combination with other remedies, more especially in those, in which the digitalis may have failed. *Gamboge* may be rendered perfectly mild, by triturating it with saline laxatives and diuretics. He then closes his catalogue of remedies, with some observations on the operation of *paracentesis*, upon which he observes, that it will not be expected that he should be one of its advocates, when the means he recommends, will, in almost every instance, readily remove the water; he does however acknowledge, that there are some cases in which it should be performed. We are next favoured with some remarks for the regulation of the diet; in general, we are told, such food should be selected which contains the greatest quantity of nutriment in the smallest bulk; thus flesh of mature animals should be preferred to that of young ones, roasted meats to boiled; vegetables should be sparingly used, except those that are *farinaceous*, as rice, which may be fully allowed. With a series of such observations, the subject of cure is dismissed, and would we could say, that part of the work concluded; but the reader has yet to pass through a section consisting of thirty-six pages, upon the *modus operandi* of Foxglove.—We shall abstain from giving an analysis of his opinions, as they are purely speculative, and possess but little novelty; true to the cause of Cullen, he contends for the direct sedative operation of this medicine, and denies altogether the stimulant effect attributed to it by the partizans of the Brunonian school. The author appears indifferent, as to the fate of the speculative opinions he has advanced, and observes, that whether they be confirmed or refuted, the practical part of his Essay will not be invalidated.

We wish he had not so far mistaken his road, as to have quitted the sober and steady path of experience, to range in a field of doubt and speculation; we must be excused for expressing our disapprobation of it, and our regret at his not having left the subject to the pen of other logicians. Upwards of a hundred cases compose an Appendix, which illustrate his peculiar plan of treatment, and the success of it.

Thus terminates the work which we have endeavoured to analyse; and we trust, our readers will coincide with us in considering, that the practical information it imparts is of considerable importance; and although we must confess, that the confidence with which he avows the superiority of his treatment, inspired us with hopes of success beyond what the perusal of his work has realized, or, indeed, the nature of the disease could render possible; yet, we must acknowledge that his plan is judicious, and the effect of it successful. The style of the writing is, upon the whole, well adapted for a practical essay; perspicuity is studied rather than embellishment, and although by a more judicious selection of words, the feeble and inharmonious period would often become more energetic and pleasing, yet we seldom find a passage whose language is distressing to the ear, or whose meaning we do not at once comprehend.

---

### *Annual Report of the Royal Humane Society*, 1809.

It is pleasing to contemplate the beneficial effects resulting from the exertions of this laudable Institution, there having been no less than 182 claimants for its rewards last year, 118 of which were cases of imminent danger restored to health, or two succesful cases at least in every three.

The volume commences with a neat tribute to the memory of the late Dr. Hawes, whose ardent zeal and indefatigable perseverance are well known, and who may justly be considered the Founder of this Society. It appears from the report here given, that the Doctor first took the idea of establishing this Society from the accounts published of a similar institution in Holland; and as many persons may be gratified by a detail of the humble origin of this now flourishing body, we shall transcribe the whole passage.

"Holland, being intersected by numerous canals and inland seas, its inhabitants were, consequently, much exposed to accidents by water; and many persons were drowned from the want of proper assistance. Hence, in the year 1767, a Society was formed at Amsterdam, which offered premiums to those who saved the life of a citizen in danger of perishing by water: it proposed to publish the methods of treatment, and to give an account of the cases of recovery. Instigated by this example, the Magistrates of Health at Milan and Venice issued orders, in 1768, for the treatment ment of drowned persons. The city of Hamburgh appointed a similar ordinance to be read in all the churches, extending their succour, not merely to the drowned, but to the strangled, to those suffocated by noxious vapours, and to the frozen. The first part of the Dutch memoirs was translated into the Russian language, by command of the Empress. In 1769 an edict was published in Germany, extending its directions and encouragements to every case of apparent death, which afforded a possibility of relief. In 1771, the Magistrates of the city of Paris founded an institution in favour of the drowned, &c. And the repeated instances of success in each country abundantly confirmed the truth of the facts related in the Amsterdam memoirs. These memoirs were, in 1773, translated into English by Dr. Cogan, in order to convince the British Publick of the practicability, in many instances, of recovering persons who were apparently dead, from drowning. No sooner were they translated than they engaged the humane and benevolent mind of Dr. Hawes. His very soul was absorbed with the animating hope of saving the lives of his fellow-creatures: but, in making the attempt, he had to encounter with ridicule and opposition. The practicability of resuscitation was denied. He ascertained its practicability, by advertising to reward persons, who, between Westminster and London bridges, should, within a certain time after the accident, rescue drowned persons from the water, and bring them ashore to places appointed for their reception, where means might be used for their recovery, and give immediate notice to him. Many lives were thus saved by himself and other medical men, which would otherwise have been lost. For twelve months he paid the rewards in these cases; which amounted to a considerable sum. Dr. Cogan remonstrated with him on the injury which his private fortune would sustain from a perseverance in these expences; he therefore consented to share them with the public. They accordingly agreed to unite their strength, and each of them to bring fifteen friends to a meeting at the chapter Coffee-house, with the express intention of establishing a Humane Society in London: this was happily accomplished in the summer of 1774. The objects of this Society was then, like that at Amsterdam, confined to the recovery of persons who were apparently dead from drowning."

Various were the obstacles the first promoters of this benevolent plan had to encounter, and the striking novelty of it did not in this instance interest the public so strongly in its favour as is usually the case; perhaps there may be much truth in the following remark; for though we are very far from being inclined to attribute selfish motives to the supporters of our numerous public charities, yet it must be acknowledged, that the gratification which arises from bestowing a personal favour on distressed objects, has now and then no inconsiderable weight.

"There was another obstacle to the rapid success of our Society. In other institutions the subscribers have the means of affording fording relief to some sick or distressed neighbour; they have a something at their own disposal; some good they can personally confer, when an application is made to them for that purpose. We have nothing of the kind; we have only an Anniversary Sermon to present to you, and this Annual Report."

As usual, directions are given for the treatment of persons apparently dead, and a description of the Society's apparatus is subjoined. Among the engravings contained in this volume is one of a portable bed, an ingenious contrivance of the Rev. Mr. Davies, of Leicester, the object of which is to afford a general warmth to the whole body, and it seems very well adapted to the purpose, and must prove extremely useful when employed in cases of suspended animation. The Society have also in the directions for prevention of premature death, turned their attention to several subjects, which though not strictly coming within their original plan, are yet highly important in themselves, such as the method of preventing the effects of lightning; the fatal effects of drinking cold water when a person is warm; the effects of excessive cold; the danger from exposure to the excessive heat of the sun; and the burning of the clothes of females; upon all of which subjects any remarks are unnecessary. Among the cases of recovery, is an excellent one, communicated by Mr. Addington, in which the progressive symptoms from a state of apparent death to complete recovery, are well described. Bleeding from the arm was employed, seemingly with every good effect, and Mr. Addington has subjoined some judicious remarks, in which he contends for the utility of that remedy, and endeavours to controvert the opinion of those who consider debility as the chief source of danger in these cases. Much attention has lately been paid to the symptoms of various diseases, with a view of ascertaining what share the brain has in producing them, and whether debility is so frequent a concomitant of diseases, as has been supposed; and as this question is likely to come before us in a variety of shapes, we shall embrace a future opportunity of offering a few remarks on this important subject. A List of the Officers and Members of the Society closes this interesting little volume.

---

*A Series of original Experiments on the Foot of the living Horse, exhibiting the Changes produced by Shoeing, and the Causes of the apparent Mystery of this Art.* By Bracy Clark, Veterinary Surgeon, F. L. S. &c. 4to. London, 1809.

The importance of the horse to mankind, in war and the chace, was known in the earliest ages, and his uses have so much increased with the advancement of civilization and the refinements of luxury, that man may now be considered as a real Centaur. Without the strength, swiftness and docility of the horse, he can neither defend himself from his enemies, procure his necessaries, or preserve his health. And yet it has somehow unaccountably happened, pened, that this noble, grateful, and generous animal, both in health and disease, has till very lately been committed to the care of persons the most unqualified for such a task. It is true, that the wants of the horse are very few; and his diseases, as never brought on by folly or intemperance, are therefore but few. Since, however, the real value and importance of his services, and the *present* shortness of his serviceable life, have been fully appreciated, men of science and education have been encouraged to devote their time and attention to his health and preservation.' Our author may justly be ranked in this class. He has only published the first part of his intended work at present; in which, he explains the structure, anatomy, and uses of the several parts of the horse's foot, when completely formed, and in a state of Nature. He then relates his experiments made for several years on the living horse, in order to demonstrate the changes produced on the foot by the present method of shoeing, and the consequences of those changes in shortening the useful life of the animal. As these explanations must necessarily be made the foundation of his future reasonings, and plans for obviating those evils and prolonging the services and comforts of a servant so valuable, he has been particular in describing the structure and uses of the several parts of the foot. This has obliged him, in a few instances, to coin a new term, or limit the use of an old one; and indeed, so beautifully are all the parts adapted to each other, and the whole to the speed, weight and strength of the animal, that we can assure the philosophical reader, that he will be well repaid for the time he employs in acquiring a correct idea of the horse's foot, in a state of nature. To persons totally unused to anatomical descriptions, the subject will probably appear complicated; but we think the force of the author's reasoning, and the truth of his conclusions, cannot be properly understood, without a knowledge of the structure and uses of those parts which he has accurately described in the beginning of the book. These are, the heels, the wall or hoof, the frog, the sole, the horny heels, the bearings of the hoof on the ground, the wear of the hoof, and the cartilages of the foot. The description of these parts is assisted by plates, but the stile is so condensed in general, that we apprehend some readers will think the subject difficult to be understood. The author concludes this anatomical and physiological part of his book with stating,

" Such appear to be the leading principles of construction in the foot of the horse in each separate part, and in the whole combined, as far as our humble reflections and researches have enabled us to consider them. It is these principles, when rightly understood, that can unfold the obscure and intricate effects of the shoe, and these alone: for the shoe, from its nature, cannot in any respect participate in these properties of the foot, and hence the cause of its mischievous effects.

" The assertion, at first, may appear singular to those who have not investigated these matters with a close attention, or viewed the chain chain of connexion of these things from the beginning of the services of the animal to his termination at the slaughter-house, through the different periods of his rapidly destructive course; but it is nevertheless true, that the shoeing the animal is, with its multifarious train of consequences, that for the most part has been the root of so many evils to the horse and to mankind, not only by its immediate operation on the structure of the foot, but by its entailed consequences in the use of him, that he is so often rendered unsatisfactory, vexatious, and dangerous through it: for these errors in the management of the feet are ever visited with unmerited punishment upon the animal himself, in order to do away or overcome its consequences by exciting other feelings, though for the most part in vain; and it is from this that the vehicles for draught are filled with all our best saddle horses, setting aside all considerations of humanity, which for certain reasons we purposely exclude from this part of our labours; and it may be with truth averred, that such is the simple nature of the animal himself, and his disorders, exclusive of the shoeing and its effects, that there would be little room for the exercise of *knowingness* or trick respecting him by stable-men or others, if these effects were fully understood, or could in any way be removed; and the dread many have for very good reasons of using horses, or having to do with them at all, would in a great degree be done away.

"For let whatever will be said about these effects being known of the shoe, it is clear from the readiness with which people consent to have their horses shod at any age, on the first summons of the breaker-in of the horse, that they view the shoe merely as protecting the foot, and are not aware of its insidious effects; nor do they afterwards exhibit the least jealousy or anxiety about it, but would rather, as we often observe, treat the proposition of its removal as a piece of inhumanity.

"And we derive some consolation from one reflection on this subject, that even with a continuance of the present method of shoeing, a considerable share of the evil may be removed, and the rapidity of the destruction be greatly in all cases prolonged under certain regulations, though we must admit also there are some services or situations in using the horse, that do not appear to allow of much assistance: but this will be made the subject of future consideration.

"If what we have stated respecting the nature of the horse's foot be true, the effect of the shoe will be almost presumed without any demonstrative evidence; but though reasoning may easily err, and imagination lead us astray, still the actual experiment, if truly related, will ever stand as plain matter of fact, that can neither err nor be denied; and the course of the experiment will also unfold a variety of matter for reflection respecting the foot, which would not properly attach to any thing we have heretofore noticed. We proceed, therefore, to the consideration of this first experiment on the effects of the shoe, in which the public, as far as as they are interested in this important inquiry, and especially myself, are greatly indebted to the obliging conduct of Mr. George Hobson, both in providing the subject, and in allowing the mare on all proper occasions to be brought for examination, and the prosecution of these experiments; for next in every state to man himself, in public utility, will be what respects the services and true knowledge of this animal, and how we can best obtain and prolong the period of his services."

The experiments by which Mr. C. demonstrates the pernicious effects of shoeing upon the feet of the horse, consist in taking casts with plaster of Paris, repeating them at proper intervals, and then comparing them with each other. These afford the particulars of the change that takes place in the size and form of the foot, upon which the subsequent tenderness and lameness principally depend. Other circumstances unfolded themselves to him, as the experiment proceeded, which were not foreseen or expected. We shall present the reader with a few of the details.

"A young mare of great beauty, and turned of five years old, was brought to my shoeing forge from Weymouth Mews, to be shod, that had been bred by George Hobson, Esq. and permitted to run wild and unshod till her fifth year, that her strength and growth should be as much as possible compleated before she was brought into use. The opportunity so extraordinarily afforded me of making the expeirment was not to be lost: for a second, I thought, might not occur; and such another has in reality never occurred to this day. Timid, and unused to have her feet meddled with, to get an impression was attended with some difficulty; the plaster of Paris was poured upon her foot held sole upwards; but before it could well set she grew uneasy at the position, and dashing her foot to the ground, broke it in a thousand pieces, and a second also in the same way. After this, as might be expected, she grew more impatient at being handled, and I almost despaired of succeeding. Being surrounded by many persons, I hoped to effect it better if she was led alone to the stable; and giving her a feed of corn, the better to take off her attention, I placed the foot, unperceived by her, in a bowl containing plaster wetted with warm water, that it might set the more readily. After waiting a few minutes, and the plaster had become perfectly hard, I drew it away from the foot without much difficulty, and it exhibited a complete impression of her foot in all its circumstances. This was done on the fourth day of June 1804.

"After smearing this impression, or mould, with a little lard, to prevent adhesion, some fresh plaster was cast upon it; I thus obtained the figure of the foot represented in *Plate* I; and for the beauty and symmetry of its parts, nature perhaps does seldom surpass it.

"That the reader who is not much used to the study of horses may make himself acquainted with the parts of the horse's foot, we shall, says Mr. C. here describe them in a general manner. They are are given for this purpose as large as in nature, that there might be less possibility of error; for the natural horse's foot has never, I believe, before been very truly represented; and by doing this he will be the more prepared to trace the changes it is doomed to undergo by artificial aid. The representation has been admitted, both by the draftsmen and engraver, to be attended with difficulty; and, but for the kind assistance of my very worthy and ingenious friend Mr. Sydenham Edwards, it would not have been nearly so well represented as it is; we may also remark, that a tolerably distant view of it, from its being so large, makes it appear to more advantage than a nearer one."

The description of the several parts then follows, for which, as well as the plates, which are necessary to illustrate them, we must refer to the work itself.

Exactly a year and nine days after the first application of the shoe, a second cast was taken from the same foot, of which an engraving is given. "During the whole of this period the shoeing smiths, who were as steady men to the full as any others in their line, were left to the practice of their own art, without the smallest interference or control of any kind on my part. They were aware of the cast being taken from the foot, and were not less careful on that account in their attentions in shoeing her."

"Let us now," says the Author, "mark with precision the differences which have taken place, and see what have been the effects of fixing the foot without intermission for a period of twelve months to an inflexible iron ring, for such is briefly the fact with respect to the nature of the shoe, by whatever name it may be called; for the word *shoe* has had its fascination also in concealing its effects, by reminding us of the comforts we derive from our own shoes made of leather, and elastic to the foot, to which neither in the material of which it is made, or the mode of its application, has it the smallest correspondence; of such force are names that mere chance often confers on things in blinding our views of their actual nature.

"The original state and proportions of the foot being before us, and perfectly preserved, we are enabled to make an exact comparison of its former and present condition; a diminution of volume throughout is strikingly manifest, but more so in the elastic parts. A mechanical hardness marks the appearance of it, in place of the flowing easy outlines observable in the original cast. The evident competency in the parts to their respective offices, which the eye recognizes in the former, is done away in this; and such is the general diminution of the foot, that actual lameness would naturally be supposed the effect of so much alteration unless explained, for this does not take place for the following reasons, — that the parts have suffered their alterations slowly, and, from being in their nature yielding and elastic, have given way to the effect of he shoe, as far as the diminution extends at present, without much esistance; and above all, that during the application of the shoe the the parts that have most suffered are not called into action, nor are their uses required, so that the foot by degrees assumes a new sort of existence, and gradually adapts itself, as much as a living part can, to the effects of the iron circle, and cannot afterwards do without it.

"We now examine the nature and extent of these changes wrought by the shoe; first observing, that in drawing away this second impression we were surprised to find with how much greater force it was held, and difficulty it came away from the foot, than the former cast did, and as immediately appeared from certain alterations that had taken place in the relative situation of the parts of the foot, as also from the slanting surfaces of the bars and frog having assumed a more perpendicular direction.

"The elastic parts of the heels have lost their swelling, rounded, and beautiful appearance, by the sinking of the cartilages and the loss of the elastic matter within; and the surface they now present is an ugly flat slope towards the base of the frog.

"The horny heels, from one to the other, in the original state of the part, measured somewhat more than four inches; in the second cast scarcely three. The foot, measured across its widest part, viz. at the greatest swell of the quarters, was in the original cast nearly five inches and a half; in the second cast it was four inches and seven-eighths. The actual length of the foot, we may remark, has not been much changed; which seems to confirm the circumstance that the cause operating these effects had been lateral principally, and serves to evince its having been the effect of the nails.

The frog had lost, through its being wasted and cut away by the smiths, the rounded swelling and projection we have distinguished by the name of the *Cushion*; and its lower surface, though its substance was so diminished, was lower by near one fourth of an inch than the horny heels, or wall; for it may be recollected, in the account we gave of the frog, that this part was then three-eighths of an inch within this level. This appeared to arise in part from the condensation of the horn of the heels, from the constant pressure of the shoe upon them, and also from the circumstances we have before explained, on the cause of this descent of the frog, which it will be unnecessary to repeat. The texture of the frog, from an agreeable yielding and elasticity, had become hard and unyielding to any impression of the fingers; and its sides, which at first were gently inclining or sloping to the commissure, had become almost perpendicular. The *cleft* at the base of the frog had become partly closed, forming a rounded ill-formed hole, and much deeper than the cleft of the natural foot. The base of the frog, which was in the natural foot of the width of two inches and a half, had now become hardly so much as two inches. The bars had considerably lost their sloping direction, and had become more perpendicular and encroaching upon the sides of the frog, and consequently more disposed to compress it.

"The

"The sole appeared somewhat more arched or cupped than formerly, but the degree of thickening it had undergone, as also the elasticity it had lost could not be accurately ascertained in the living subject. Thus we see the beautiful and useful symmetry of nature's mould, no part of which is without its use, has been changed by artificial restraint to deformity and incompetence. Many there are who have added unnecessarily to the obscurity of these cases by confounding them with, or supposing them the effects of standing in the stable; of which, in the next part, to set things in a more clear light, we shall give proof enough from actual experiment, that however inimical to the feet the stable may be, it is wholly incapable of producing such powerful effects as these, which can be shown most convincingly in two ways; viz. by shoeing, and turning the horse to grass, when the same effects will ensue; and also by keeping a horse unshod in the stable, which we have for years done, when no effects of this kind have taken place. The worst cases of contraction also, we may observe, are with stage horses, that have little standing in the stable.

" No shoeing-smith or dealer would complain of the foot as it appears in fig. 2; though it is a wide departure from the model which nature has established for the foot of the horse. And so little has this fact been attended to, even by those better informed than dealers or smiths in these matters, that I remember some years back Mr. St. Bel, the first professor of the veterinary college, sending forth to the world with his Essay on Shoeing, as a model of a perfect foot of the horse, one more diminished than this; nor did he know that nature, from the use of the shoe, had suffered much change; nor did any of us studying at that time at the college at all suspect it, at least, that there was in these cases much alteration of an injurious nature.

" If horses were brought there with contracted feet, as must have been daily the case, they scarcely obtained notice, from our habits of constantly seeing them in this state, unless they were attended with very great crippling and tenderness indeed; for it is most usual with people finding defects of this sort to avoid the evil by parting with their horses, and to take as little notice of the fact as possible; and many there are who stoutly deny there is any such thing as tenderness in the fore feet of horses; and some there are who appear insensible of it till a fall convinces them of their danger, when they are apt to become as much too timid as before they were too confident. Horses of this description, it has been before stated, can still, after they are sold, be made serviceable; for employ will never be wanting for the cheap horse as long as severe bits and a bearing rein can keep them up, or the thong can draw from them an exertion, for harness is the only resource while they last. And these defects of the feet were somehow or other considered as casually arising from a defective nature of the foot itself, or from bad shoeing, as it was termed: and in one sense this might be true; for if shoes were fitted out very small with a view to to *neatness*, as it is called, it would obviously accelerate the mischief in a greater degree than when a more liberal allowance was made in this respect, and which circumstances resting entirely with the discretion of the workmen, in no way, perhaps, suspicious of its consequences, would ever be a matter of uncertainty; for the fact appears to be, that the finer the feet the sooner they are destroyed: hence the feet of blood horses are the first to experience its effects.

"Five years of unrestrained growth had perfected this mare's foot beyond what is generally seen at the commencement of shoeing, which usually takes place on the second, third, or fourth year, and before the foot is nearly unfolded or grown to its size; so that the great change that is here observable is more strongly manifested than it would be in ordinary cases of shoeing; and the foot cannot be expected to exhibit differences so great and conspicuous in succeeding years as in the first, there being less of elastic matter to act upon. Nevertheless, every year will have its effects, and will bring the hoof in closer approximation to the coffin-bone; and at length we shall see that a partial diminution of the bone itself will be the consequence, with other derangements of it.

"The horse, we may remark, like other large animals, is slow in acquiring maturity, and like them, is not very short-lived. Some celebrated writers have considered the natural period of his life about fifty years. This was before the art of shoeing commenced, and may be not far from the truth in those times. If we were to give an opinion on this matter, we should state it as our belief, that he acquires his stature or height at about five years, but obtains his full bulk and strength about the eighth year; and this period, as in most other animals, if multiplied by four, will give somewhere about the period of his natural life; which, without any desire of unnaturally extending, would be from thirty-two to forty; and at the former age we have seen (setting aside the state of his feet) horses capable of a great deal of service. But what we wish to remark is, that frequent visits to the slaughter-house, a useful school, but not much frequented, have led us to observe and conclude, that six arrive there before fourteen to [illegible]e after the fourteenth year! for they so early become cripples through the injuries of their feet, that it is found most advantageous to the interests of those who get these kind of horses that are daily becoming tenderer, to "use them up" by the severest measures, and most unnatural usage, rather than to endeavour to prolong their labours by preserving them; and there is no want of supply through the causes above described, at least principally; and it deserves a closer attention from the public than it has ever yet received; for men, as we have before observed, have been really afraid to look into these things about horses, as though their affairs were somehow clothed in fearful and impenetrable mysteries."

Knowing the true cause of this destruction of the feet, it is amusing to hear the opinions of those, to whose care this noble animal has been committed for so many centuries.

"If the stable-keeper is asked why his horses are so tender footed before? and why there needs so much trouble to keep them up? so much so, that all pleasure in riding is destroyed, his answer is, 'Why, horses to be sure will by use become leg-weary, and every one who *knows any thing about horses* knows that well enough,' and with a smile at the simplicity of the enquirer he quits the subject.

"If any one, not having the usual awe of this character, should ask the coachman why he wants two or three kinds of irons to be put in his horse's mouth, his answer will be, 'Why, would any one be so mad as to attempt to drive without them?' Then if you are apprehensive of your horses' falling, what is the cause of this? 'Go ask the smiths, they can tell you better about it, they don't *shoe them safely*.'

"If the shoeing smith be enquired of respecting this matter, and how does the horse become tender? 'Why, it is to be sure from always standing on the dry litter of the stables, and that is plain enough, for the hind feet are never affected, because they are more in the dung and moisture, which makes it clear enough;' and thus this business is disposed of without further trouble among them."

Our author is still continuing his experiments on the same mare, and has given an engraving of a cast taken after two years shoeing, and another after three years. By a comparison of these with that representing the foot in its natural state, the most superficial observer cannot fail to notice the great change in the outline of its form. In the natural state, the bearing part is nearly circular; a figure which is well known to contain the greatest possible area, and consequently the greatest bearing under the same circumference. Every mechanic would therefore infer, that this was the best possible form for the sole of a quadruped of such weight, strength, and swiftness. In the feet that have borne the use of the shoe for only three years, the circular form is changed into one almost resembling a parabola.

The breadth from outside to inside is greatly diminished, while the length from heel to toe is considerably increased; notwithstanding the frequent paring and filing away of the toe by the smith. As scarcely any persons except smiths, have opportunities of seeing the foot in its natural state, and they but rarely; and as the feet of the most admired horses of the blood kind, which have been shod a few years, are of this long and narrow form, we by habit learn to consider it as the most beautiful and natural.

The second part of this work, which is ready for the press, will contain an account of experiments made on other horses; plans and measures for preventing the evils resulting from the present mode of shoeing; how the *founder* in horses is produced; the defences fences of the feet used by the ancients, with a variety of other subjects mentioned in the contents which accompany this first part.

We hope this short analysis will give our readers an idea of the great merit and importance of this work; and we are confident that no veterinary surgeon who learns that it is published, will be slow in procuring a perusal of it.

